# Supplementary material for: Prevalence of persistent SARS-CoV-2 in a large community surveillance study
Source: Nature. 2024 Feb 21;626(8001):1094–101. doi: 10.1038/s41586-024-07029-4 (PMC10901734; doi:10.1038/s41586-024-07029-4)
Supplement: Supplementary file 1 — This file contains information about the number of recruited participants in the ONS COVID-19 Infection Survey (ONS-CIS) and the timing of asessments. [file 41586_2024_7029_MOESM1_ESM.docx]

**Supplementary Information**

This file contains information about the number of recruited participants in the ONS COVID-19 Infection Survey (ONS-CIS) and the timing of asessments.

**General information about recruited participants in ONS-CIS**

**(a) Status of all participants recruited in ONS-CIS.**

Status |

(UK-wide) | Freq. Percent Cum.

------------+-----------------------------------

Active | 426,334 79.58 79.58

Withdrawn | 70,689 13.19 92.77

Completed | 38,728 7.23 100.00

------------+-----------------------------------

Total | 535,751 100.00

**(b) Status of all participants recruited in ONS-CIS per country.**

Status |

Country | England Wales NI* Scotland | Total

------------+-------------------------------------------+----------

Active | 357,003 22,708 12,449 34,174 | 426,334

(Percent) | (79.14) (82.88) (83.97) (80.51) | (79.58)

------------+-------------------------------------------+----------

Withdrawn | 59,741 3,427 1,593 5,928 | 70,689

(Percent) | (13.24) (12.51) (10.74) (13.97) | (13.19)

------------+-------------------------------------------+----------

Completed | 34,336 1,263 784 2,345 | 38,728

(Percent) | (7.61) (4.61) (5.29) (5.52) | (7.23)

------------+-------------------------------------------+----------

Total | 451,080 27,398 14,826 42,447 | 535,751

(Percent) | (100.00) (100.00) (100.00) (100.00) | (100.00)

**(c) Status of all participants based on whether they died or not after being recruited.**

Status | Died

(UK-wide) | No Yes | Total

-----------+----------------------+----------

Active | 426,334 0 | 426,334

| 100.00 0.00 | 100.00

-----------+----------------------+----------

Withdrawn | 69,486 1,203 | 70,689

| 98.30 1.70 | 100.00

-----------+----------------------+----------

Completed | 37,711 1,017 | 38,728

| 97.37 2.63 | 100.00

-----------+----------------------+----------

Total | 533,531 2,220 | 535,751

| 99.59 0.41 | 100.00

**(d) Status of all participants based on whether they died or not after being recruited per country.**

|

Died | England Wales NI* Scotland | Total

-----------+--------------------------------------------+----------

No | 449,021 27,239 14,825 42,446 | 533,531

Percent | 99.54 99.42 99.99 100.00 | 99.59

-----------+--------------------------------------------+----------

Yes | 2,059 159 1 1 | 2,220

Percent | 0.46 0.58 0.01 0.00 | 0.41

-----------+--------------------------------------------+----------

Total | 451,080 27,398 14,826 42,447 | 535,751

Percent | 100.00 100.00 100.00 100.00 | 100.00

**(e) Number of participants recruited through the ONS-CIS in 2022.**

|

Date | England Wales NI* Scotland | Total

-----------+--------------------------------------------+----------

02jan2022 | 96 0 3 0 | 99

03jan2022 | 108 0 6 8 | 122

04jan2022 | 90 0 2 14 | 106

05jan2022 | 55 0 0 6 | 61

06jan2022 | 58 0 0 10 | 68

07jan2022 | 64 0 3 2 | 69

08jan2022 | 83 0 0 9 | 92

09jan2022 | 42 0 4 0 | 46

10jan2022 | 62 1 4 7 | 74

11jan2022 | 41 0 0 7 | 48

12jan2022 | 43 0 0 8 | 51

13jan2022 | 27 0 2 11 | 40

14jan2022 | 52 2 1 1 | 56

15jan2022 | 29 0 0 5 | 34

16jan2022 | 20 0 0 0 | 20

17jan2022 | 19 0 0 0 | 19

18jan2022 | 13 0 0 2 | 15

19jan2022 | 17 0 0 11 | 28

20jan2022 | 29 0 1 2 | 32

21jan2022 | 12 0 0 0 | 12

22jan2022 | 19 0 0 1 | 20

23jan2022 | 14 0 0 0 | 14

24jan2022 | 11 0 0 0 | 11

25jan2022 | 7 0 0 2 | 9

26jan2022 | 10 0 0 0 | 10

27jan2022 | 1 0 0 0 | 1

28jan2022 | 0 0 0 2 | 2

29jan2022 | 5 0 0 3 | 8

04apr2022 | 1 0 0 0 | 1

04may2022 | 1 0 0 0 | 1

19oct2022 | 0 0 1 0 | 1

20oct2022 | 0 0 4 0 | 4

21oct2022 | 0 0 1 0 | 1

22oct2022 | 0 0 1 0 | 1

24oct2022 | 0 0 3 0 | 3

26oct2022 | 0 0 1 0 | 1

09nov2022 | 0 0 3 0 | 3

12nov2022 | 0 0 0 1 | 1

18nov2022 | 0 0 1 0 | 1

-----------+--------------------------------------------+----------

Total | 1,029 3 41 112 | 1,185

*NI = Northern Ireland

**General information about the number and timing of assessments**

**(a) All assessments between 2 Nov 2020 and 15 Aug 2022 inclusive with swab test result (positive/negative/void).**

Result | Freq. Percent Cum.

--------------------+-----------------------------------

Negative | 7,962,852 96.20 96.20

Positive | 155,742 1.88 98.08

Void | 158,719 1.92 100.00

--------------------+-----------------------------------

Total | 8,277,313 100.00

**(b) Number of days since last assessment. Columns with pi represent the i^th^ percentile of the frequency distribution (number of days).**

Results | N p50 p25 p75 p5 p95 p1 p99 Min Max Mean SD

-----------------+------------------------------------------------------------------------------------------------------------------------

Negative | 7,736,259 28 26 34 7 62 6 101 0 734 31.76518 18.36857

Positive | 153,220 30 27 37 10 68 6 112 0 639 35.17064 20.05956

Void | 155,012 29 27 35 7 65 6 108 0 584 33.15867 19.3363

-----------------+------------------------------------------------------------------------------------------------------------------------

Total | 8,044,491 28 26 34 7 62 6 101 0 734 31.85689 18.4281

------------------------------------------------------------------------------------------------------------------------------------------

**(c) Number of days to the next assessment.**

Results | N p50 p25 p75 p5 p95 p1 p99 Min Max Mean SD

-----------------+------------------------------------------------------------------------------------------------------------------------

Negative | 7,812,232 28 27 35 7 66 6 115 0 739 33.03203 20.49527

Positive | 150,697 31 28 38 12 78 6 131 0 531 37.08509 22.81872

Void | 155,675 29 27 35 7 65 6 113 0 575 33.21803 19.70951

-----------------+------------------------------------------------------------------------------------------------------------------------

Total | 8,118,604 28 27 35 7 67 6 115 0 739 33.11083 20.53333

------------------------------------------------------------------------------------------------------------------------------------------

**(d) Number of days since last assessment for individuals with positive swabs.**

positive | N p50 p25 p75 p5 p95 p1 p99 Min Max Mean SD

---------+------------------------------------------------------------------------------------------------------------------------

Ct<30 | 109,194 30 28 38 14 68 6 111 0 488 35.57288 19.85713

Ct>=30 | 42,663 29 27 36 7 66 6 111 2 639 33.92024 20.39259

Ct NK | 1,363 35 30 42 28 87 20 125 6 273 42.08511 22.78157

---------+------------------------------------------------------------------------------------------------------------------------

Total | 153,220 30 27 37 10 68 6 112 0 639 35.17064 20.05956

----------------------------------------------------------------------------------------------------------------------------------

**(e) Number of days to the next assessment for individuals with positive swabs.**

positive | N p50 p25 p75 p5 p95 p1 p99 Min Max Mean SD

---------+------------------------------------------------------------------------------------------------------------------------

Ct<30 | 107,301 31 28 38 20 78 6 131 0 531 37.38413 22.58054

Ct>=30 | 42,042 30 27 38 7 80 6 132 1 426 36.41278 23.67145

Ct NK | 1,354 33 28 39 22 50 18 76 6 183 34.26219 10.86591

---------+------------------------------------------------------------------------------------------------------------------------

Total | 150,697 31 28 38 12 78 6 131 0 531 37.08509 22.81872

----------------------------------------------------------------------------------------------------------------------------------

**(e) Number of days to the next assessment of individuals with persistent infections (N=381) and reinfections (N=60) identified in our study.**

reinfection | N p50 p25 p75 p5 p95 p1 p99 Min Max

------------+----------------------------------------------------------------------------------------------------

Reinfection | 60 29 28 32 26 43.5 16 52 16 52

Persistent | 381 29 27 32 26 42 22 55 8 64

------------+----------------------------------------------------------------------------------------------------

Total | 441 29 28 32 26 42 22 54 8 64

-----------------------------------------------------------------------------------------------------------------
