# Supplementary material for: Prevalence of persistent SARS-CoV-2 in a large community surveillance study
Source: Nature. 2024 Feb 21;626(8001):1094–101. doi: 10.1038/s41586-024-07029-4 (PMC10901734; doi:10.1038/s41586-024-07029-4)
Supplement: Supplementary file 2 — Reporting Summary [file 41586_2024_7029_MOESM2_ESM.pdf]

nature portfolio

Corresponding author(s): Mahan Ghafari and Katrina Lythgoe

Last updated by author(s): Nov 28, 2023

Reporting Summary

Nature Portfolio wishes to improve the reproducibility of the work that we publish. This form provides structure for consistency and transparency in reporting. For further information on Nature Portfolio policies, see our Editorial Policies and the Editorial Policy Checklist.

Statistics

For all statistical analyses, confirm that the following items are present in the figure legend, table legend, main text, or Methods section.

n/a	Confirmed	
☐	☒	The exact sample size (*n*) for each experimental group/condition, given as a discrete number and unit of measurement
☐	☒	A statement on whether measurements were taken from distinct samples or whether the same sample was measured repeatedly
☐	☒	The statistical test(s) used AND whether they are one- or two-sided<br>*Only common tests should be described solely by name; describe more complex techniques in the Methods section.*
☐	☒	A description of all covariates tested
☐	☒	A description of any assumptions or corrections, such as tests of normality and adjustment for multiple comparisons
☐	☒	A full description of the statistical parameters including central tendency (e.g. means) or other basic estimates (e.g. regression coefficient) AND variation (e.g. standard deviation) or associated estimates of uncertainty (e.g. confidence intervals)
☐	☒	For null hypothesis testing, the test statistic (e.g. *F*, *t*, *r*) with confidence intervals, effect sizes, degrees of freedom and *P* value noted<br>*Give P values as exact values whenever suitable.*
☒	☐	For Bayesian analysis, information on the choice of priors and Markov chain Monte Carlo settings
☒	☐	For hierarchical and complex designs, identification of the appropriate level for tests and full reporting of outcomes
☒	☐	Estimates of effect sizes (e.g. Cohen's *d*, Pearson's *r*), indicating how they were calculated

Our web collection on statistics for biologists contains articles on many of the points above.

Software and code

Policy information about availability of computer code

Data collection	No software was used for data collection.
Data analysis	For consensus sequence construction, we used shiver (v1.5.8); ARTIC Nextflow processing pipeline (v1)<br>For phylogenetic analysis, we used IQ-TREE (v1.6.12); TempEst (v1.5.3); ggtree (v3.6.2)<br>For calculation of p-values and visualisation of histogram and box plots, we used Mathematica (v13.1.0.0)

For manuscripts utilizing custom algorithms or software that are central to the research but not yet described in published literature, software must be made available to editors and reviewers. We strongly encourage code deposition in a community repository (e.g. GitHub). See the Nature Portfolio guidelines for submitting code & software for further information.

Data

Policy information about availability of data

All manuscripts must include a data availability statement. This statement should provide the following information, where applicable:

- Accession codes, unique identifiers, or web links for publicly available datasets
- A description of any restrictions on data availability
- For clinical datasets or third party data, please ensure that the statement adheres to our policy

All raw consensus sequences have been made publicly available as part of the COVID-19 Genomics UK (COG-UK) Consortium (https://www.cogconsortium.uk/priority-areas/data-linkage-analysis/public-data-analysis/) and are available from the European Nucleotide Archive (ENA) at EMBL-EBI under accession number

PRJEB37886 (http://www.ebi.ac.uk/ena/browser/view/PRJEB37886). These sequences can be accessed using their COG-UK sample title which is available in Source Data 4 for persistent infections and Source Data 5 for reinfections with the same major lineage. All post-aligned consensus sequences (aligned to Wuhan-Hu-1 reference sequence) are available on figshare to facilitate reproducibility of our findings (https://figshare.com/s/acdaf46f87e0f9874e38). All remaining data, excluding personal clinical information on participants, are available in the main text and supporting materials.

Human research participants

Policy information about studies involving human research participants and Sex and Gender in Research.

Reporting on sex and gender	Sex was determined based on self-reporting. Gender was not considered in the study design.
Population characteristics	Extended Data Table 1 includes the basic characteristics of the population. Extended Data Figure 1 includes a flow diagram of Office for National Statistics Covid Infection Survey (ONS-CIS) participants in this study.
Recruitment	The ONS-CIS is a UK household-based surveillance study in which participant households are approached at random from address lists across the UK (see Pouwels et al Lancet 2020). The survey had rolling recruitment, but in practice most recruitments occurred between September and December 2020 (see Source Data 2 in the paper and also supplementary table 4 in Vihta et al Clinical Infectious Diseases 2022).
Ethics oversight	The study received ethical approval from the South Central Berkshire B Research Ethics Committee (20/SC/0195).

Note that full information on the approval of the study protocol must also be provided in the manuscript.

Field-specific reporting

Please select the one below that is the best fit for your research. If you are not sure, read the appropriate sections before making your selection.

☐ Life sciences ☐ Behavioural & social sciences ☒ Ecological, evolutionary & environmental sciences

For a reference copy of the document with all sections, see nature.com/documents/nr-reporting-summary-flat.pdf

Ecological, evolutionary & environmental sciences study design

All studies must disclose on these points even when the disclosure is negative.

Study description	Our study identified persistent infections and reinfections with the same major lineage of SARS-CoV-2 using genomic sequence data obtained as part of the ONS-CIS. We also reported the number of symptoms and viral load dynamics for the persistent infections throughout their infection and reinfections and estimated the rate at which persistently infected individuals self-reported as having Long Covid compared to a group of non-persistently infected individuals.
Research sample	The study included 93,927 high-quality sequenced samples from the ONS-CIS, representing 90,146 individuals living in 66,602 households across the UK (see Extended Data Figure 1). To identify persistent infections and reinfections with the same major lineage, we limited the dataset to individuals with two or more high-quality sequences (corresponding to RT-PCR positive samples with cycle threshold ≤30), taken at least 26 days apart, and where consensus sequences were of the same major lineages of Alpha, Delta, BA.1 or BA.2. Description of the number of samples, age and sex of persistently infected individuals per major lineage is provided in Extended Data Table 1 and Extended Data Figure 1.
Sampling strategy	All individuals aged two years and older from each household who provide written informed consent provide swab samples (taken by the participant or parent/carer for those under 12 years), regardless of symptoms, and complete a questionnaire at assessments, which occur weekly for the first month in the survey and then approximately monthly. Participant households are approached at random from address lists across the country to provide a representative sample of the population (Pouwels et al Lancet 2020).
Data collection	From 26 April 2020 to 31 July 2022, assessments were conducted by study workers visiting each household; from 14 July 2022 onwards assessments were remote, with swabs taken using kits posted to participants and returned by post or courier, and questionnaires completed online or by telephone. Positive swab samples with cycle threshold ≤30 were sent for sequencing.<br><br>From February 2021, at every assessment, participants were asked "would you describe yourself as having Long Covid, that is, you are still experiencing symptoms more than 4 weeks after you first had COVID-19, that are not explained by something else?". When evaluating the probability of reporting Long Covid in persistently and non-persistently infected individuals, we considered the first assessment at least 12 weeks and at least 26 weeks after infection.<br><br>The survey offered participants the option of only having one enrollment assessment (taken by ~1%), or only assessments for one month (taken by ~1%; see Extended Data Figure 1).
Timing and spatial scale	For this analysis, we included data from 2nd November 2020 to 15th August 2022, spanning a period from the earliest Alpha to latest Omicron BA.2 sequences within the ONS-CIS dataset. Sample are collected from population across the UK. Status of all participants recruited in ONS-CIS per country is provided in Source Data 2.

Data exclusions	Only positive RT-PCR samples with Cycle threshold ≤30 (high viral titre) have been selected for sequencing. To ensure all sequences have high coverage, we only included sequences with >50% genome coverage.<br><br>The Long Covid analysis used complete cases, i.e. excluded those who did not have a response to the Long Covid in this timeframe (see Extended Data Figure 1).<br><br>All excluded data points are shown in Extended Data Figure 1.
Reproducibility	The original fasta files including consensus sequences of persistent infections and reinfections with the same major lineage as well as the other sequences used for the phylogenetic analysis are provided in the supplementary materials.
Randomization	Randomization was not relevant to the study design as it was based on epidemiological observations in the general population and no experimental treatment was applied.
Blinding	Blinding was not relevant to the study design as it was based on epidemiological observations in the general popukation and no experimental treatment was applied.

Did the study involve field work? ☐ Yes ☒ No

Reporting for specific materials, systems and methods

We require information from authors about some types of materials, experimental systems and methods used in many studies. Here, indicate whether each material, system or method listed is relevant to your study. If you are not sure if a list item applies to your research, read the appropriate section before selecting a response.

Materials & experimental systems

n/a	Involved in the study
☒	☐ Antibodies
☒	☐ Eukaryotic cell lines
☒	☐ Palaeontology and archaeology
☒	☐ Animals and other organisms
☒	☐ Clinical data
☒	☐ Dual use research of concern

Methods

n/a	Involved in the study
☒	☐ ChIP-seq
☒	☐ Flow cytometry
☒	☐ MRI-based neuroimaging
