# Supplementary material for: Prevalence of persistent SARS-CoV-2 in a large community surveillance study
Source: Nature. 2024 Feb 21;626(8001):1094–101. doi: 10.1038/s41586-024-07029-4 (PMC10901734; doi:10.1038/s41586-024-07029-4)
Supplement: Supplementary file 4 — Authors and affiliations for the COVID-19 Infection Survey Group [file 41586_2024_7029_MOESM4_ESM.docx]

**The COVID-19 Infection Survey team**

Tina Thomas^1^, Dawid Pienaar^1^, Joy Preece^1^, Sarah Crofts^1^, Lina Lloyd^1^, Michelle Bowen^1^, Russell Black^1^, Antonio Felton^1^, Megan Crees^1^, Joel Jones^1^, Esther Sutherland^1^, Derrick W. Crook^2^, Emma Pritchard^2^, Karina-Doris Vihta^2^, Alison Howarth^2^, Brian D. Marsden^2^, Kevin K. Chau^2^, Lucas Martins Ferreira^2^, Wanwisa Dejnirattisai^2^, Juthathip Mongkolsapaya^2^, Sarah Hoosdally^2^, Richard Cornall^2^, David I Stuart^2^, Gavin Screaton^2^, John N Newton^3^, John I Bell^4^, Stuart Cox^5^, Kevin Paddon^5^, Tim James^5^, Julie Robotham^6^, Paul Birrell^6^, Helena Jordan^7^, Tim Sheppard^7^, Graham Athey^7^, Dan Moody^7^, Leigh Curry^7^, Pamela Brereton^7^, Ian Jarvis^8^, Anna Godsmark^8^, George Morris^8^, Bobby Mallick^8^, Phil Eeles^8^, Jodie Hay^9^, Harper VanSteenhouse^9^, Jessica Lee^10^, Sean White^11^, Tim Evans^11^, Lisa Bloemberg^11^, Katie Allison^12^, Anouska Pandya^12^, Sophie Davis^12^, David I Conway^13^, Margaret MacLeod^13^, Chris Cunningham^13^

^1^ Office for National Statistics, Newport, UK.

^2^ Nuffield Department of Medicine, University of Oxford, Oxford, UK.

^3^ Office for Health Improvement and Disparities, London, UK

^4^ Office of the Regius Professor of Medicine, University of Oxford, Oxford, UK

^5^Oxford University Hospitals NHS Foundation Trust, Oxford, UK

^6^UK Health Security Agency, London, UK

^7^IQVIA, London, UK

^8^National Biocentre, Milton Keynes, UK.

^9^Glasgow Lighthouse Laboratory, London, UK

^10^Department of Health and Social Care, London, UK

^11^Welsh Government, Cardiff, UK

^12^Scottish Government, Edinburgh, UK

^13^Public Health Scotland, Edinburgh, UK
